# Tic severity and executive functioning in children and adolescents: a moderated mediation model of premonitory urges and comorbidity

**DOI:** 10.1186/s13034-025-00974-6

**Published:** 2025-11-04

**Authors:** Yanting Lu, Liangliang Chen, Duan Lin, Yajun Tang, Qinyu Li, Xiumei Liu

**Affiliations:** 1 Fujian Children’s Hospital(Fujian Branch of Shanghai Children’s Medical Center), Fuzhou, Fujian China; 2 College of Clinical Medicine for Obstetrics & Gynecology and Pediatrics, Fujian Medical University, Fuzhou, Fujian China

**Keywords:** Tic disorders, Premonitory urges, Executive function, Moderated mediation model

## Abstract

**Background:**

The severity of tics may influence executive function in children and adolescents diagnosed with tic disorders. The underlying mechanism is still inadequately researched. This study investigates the mediating role of premonitory urges in the relationship between tic severity and executive functioning, alongside the moderating effect of comorbidities.

**Methods:**

A total of 154 children and adolescents, aged 6 to 15 years, diagnosed with tic disorders, were recruited from Fujian, China. The Yale Global Tic Severity Scale (YGTSS), Premonitory Urges for Tics Scale (PUTS), and Behavior Rating Inventory of Executive Function (BRIEF) were utilized to evaluate tic severity, premonitory urges, and executive functioning. R software version 4.4.3 was used for descriptive statistics and Pearson correlation studies. The moderated mediator models were tested using Bayesian Structural Equation Modeling (BSEM).

**Results:**

A Bayesian simple mediation model revealed that the premonitory urge fully mediated the association between tic severity and executive functioning. Additionally, comorbidity was found to independently predict both the premonitory urge and executive functioning. In the context of a moderated mediation model, comorbidity intensified the association between tic severity and the premonitory urge, resulting in more pronounced indirect effects on behavioral regulation (BRI) and metacognition (MI). The Index of Moderated Mediation was significant for both BRI and MI, thereby confirming the enhancement of the mediation pathway driven by comorbidity.

**Conclusions:**

This study is the first application of BSEM to clarify the mediating mechanism through which tic severity affects executive functioning via the premonitory urge, while concurrently validating the moderating effect of comorbidities. This finding supports the optimization of clinical assessment and intervention strategies.

**Supplementary Information:**

The online version contains supplementary material available at 10.1186/s13034-025-00974-6.

## Introduction

Tic disorders, including Tourette syndrome (TS) and provisional and chronic motor/vocal tic disorders, are developmental neuropsychiatric disorders defined as repetitive, stereotypical, rapid, nonrhythmic movements or vocalizations that can be simple or complex and tend to diminish in severity over time [1]. The 2022 Epidemiological Survey of Mental Disorders in Children and Adolescents in China found that the prevalence of tic disorder was 2.5% [2]. Most patients with tic disorders experience premonitory urges, which are known as a discomfort or tension preceding their tics [3, 4]. Tic disorders are often comorbid with other disorders, including attention deficit/hyperactivity disorder (ADHD), obsessive-compulsive disorder (OCD), autism spectrum disorder (ASD), anxiety, and depression [4]. Mounting findings have revealed that tic disorders can affect patients’ cognition, quality of life, and executive functions of patients [5–7].

Executive functions refer to a set of top-down mental processes employed in the organizing, planning, and monitoring of complex, goal-directed behaviors [8]. Three core executive functions exist: inhibitory control, working memory, and cognitive flexibility, along with higher-order executive functions, including planning and problem-solving. Executive functions play a crucial role in physical and mental health; cognitive, social, and psychological development; as well as in achieving success in educational and occupational settings [9].

Although the impact of tic disorders on executive functions varies, numerous studies have shown that children with tic disorders exhibit deficiencies in a number of executive function components, including reduced working memory capacity, sustained attention and response inhibition, cognitive flexibility, and a diminished capacity for organizing and planning thoughts [10–12]. Prior studies have shown a correlation between the severity of tics and working memory and response inhibition deficits [13]. However, the mechanism underlying the effect of tic disorders on executive functions remains unclear.

Premonitory urges are considered a hallmark of tics and play a role in tic generation. Previous research has shown a significant correlation between premonitory urges and tic severity [3, 14, 15]. Moreover, premonitory urges, as a type of sensory phenomenon, are significantly associated with executive function, including the Behavior Rating Index, Emotional Regulation Index, and Cognitive Rating Index [16]. Therefore, the role of premonitory urges between tic severity and executive function warrants further investigation.

Previous studies on the association between comorbidities and premonitory urges have yielded inconsistent results. For example, a study involving 42 youths identified significant relationships between the premonitory urges and ADHD, OCD, and anxiety/depression [17]. In contrast, a separate study involving adults indicated that, after controlling for age and gender, individuals with comorbid ADHD and depression exhibited a greater likelihood of experiencing premonitory urges, whereas those with comorbid OCD reported higher intensities of these urges [3]. Studies have also shown impaired cognitive control and working memory in people with comorbid ADHD and impaired inhibition in children with comorbid OCD [12, 18–20]. A meta-analysis even found that inhibitory control was impaired in tic disorders and that co-occurrence of ADHD had an enhanced effect on inhibitory deficits in tic disorders [21]. These pathways suggest that comorbidities may actively moderate the relationship between tic severity and executive functioning by altering the strength of the premonitory urges pathway and defining distinct neurocognitive subtypes. This evidence pattern, in which specific comorbidities predicts distinct clinical and cognitive phenotypes, cannot be explained by a simple confusion model.

In this study, we aimed to investigate the relationship between tic severity and executive functioning, along with the roles of premonitory urges and comorbidities. We hypothesized that (1) the premonitory urge mediates the relationship between tic severity and executive functioning, and (2) comorbidity acts as a moderating factor in this mediating effect. This study is the first to utilize a moderated mediation model to investigate the relationship between tic severity and executive functioning, thereby potentially uncovering specific cognitive impairment pathways in tic disorders.

## Materials and methods

### Participants

The participants in the study were children and adolescents with tic disorders who were admitted to the Child Healthcare/Developmental Behavioral Pediatrics Clinic of Fujian Children’s Hospital from November 2022 to June 2024. Inclusion criteria consisted of (i) all the children met the Diagnostic and Statistical Manual of Mental Disorders, Fifth Edition (DSM-5) [1], and were diagnosed by experts in developmental and behavioral pediatrics; (ii) tic episodes occurred within 7 days before assessment; (iii) the age ranged from 6 to 16 years old, in the stage of childhood and adolescence. Exclusion criteria consisted of (i) combined with other neurological disorders (i.e. epilepsy, encephalitis, tumor, etc.); (ii) history of head trauma with loss of consciousness, ingestion of drugs that significantly alter the excitability of the cortex ; (iii) unable to understand the psychometric scale.

This study was approved by the Medical Ethics Committee of Fujian Children’s Hospital (Ethics number:2022ETKLR10036). Informed consent was also obtained from the parents or guardians of the recruited children and adolescents.

### Measures

#### Yale global tic severity scale (YGTSS)

The Yale Global Tic Severity Scale (YGTSS) is a widely used semi-structured clinician assessment tool used to evaluate the severity of motor and vocal tics over the last week. The scale has five subsections: motor tic severity; vocal tic severity; total tic severity (motor + vocal); tic impairment score; and global severity score (total tic severity + impairment score). The tic severity scores are based on the number of tics and the frequency, intensity, complexity, along with interference of the vocal and motor tics. The impairment scores are based on how the individual rates the impact of their tics on self-perception, self-esteem, personal relationships, and ability to perform in an academic/occupational setting. The score is divided into three levels: mild (< 25points), moderate (25-50points), and severe (>50points) [22].

#### Behavior rating inventory of executive function (BRIEF)

The Behavior Rating Inventory of Executive Function (BRIEF) Parent Form is a multidimensional tool for parents to assess executive functioning behaviors in children and adolescents over the past 6 months. The Chinese version of the BRIEF demonstrates high internal consistency, with a reliable test–retest of 0.68–0.89; and internal consistency of 0.74–0.96 [23]. The BRIEF consists of 86 items and assesses eight domains of executive functioning [24]. Three of these scales (Inhibition, Shift, and Emotional Control) were combined into the Behavioral Regulation Index(BRI). The remaining five scales (Initiate, Working Memory, Plan/Organize, Organization of Materials, and Monitoring) were combined into the Metacognition Index(MI). The total score of each scale was the Global Executive Composite(GEC)[25]. T-scores were calculated based on sex- and age-specific criteria, with T-scores >60 and >65 defined as potential subclinical and clinical executive functioning impairments or problems.

#### Premonitory urge for tics scale (PUTS)

The Premonitory Urge for Tics Scale (PUTS) is a self-report scale for assessing premonitory urges in patients with tic disorders. It was developed by Woods et al. A prior European study involving 656 children aged 3 to 16 years with tic disorders indicated that PUTS demonstrated strong internal reliability from early childhood through adolescence [4]. Similarly a Chinese study involving 367 typically developing children aged 6 to 16 years demonstrated strong psychometric properties across different age groups [26]. The scale contains 10 items, each rated from 1 (not at all true) to 4 (very true). It has been reported in the literature that the 10th item has a low correlation with the rest of the scale compared with the other items, so the total score is obtained by adding the scores of the 9 items. The total score ranges from 9 to 36, with higher scores indicating greater presence and intensity of the premonitory urges [27].

### Procedure

(i) We diagnosed tic disorder and its comorbidities according to DSM-5 criteria by two specialists in developmental behavioral pediatrics; (ii) The YGTSS was completed by trained developmental behavioral pediatricians using semi-structured interviews. Parents filled in BRIEF according to their children’s performance; (iii) The PUTS was completed by children with standardized assistance. For younger children, a trained physician read each item aloud and provided neutral, objective clarification using age-appropriate terms (e.g., a body feeling before a tic). After the explanation, the child subsequently selected their response. Children unable to comprehend the concept of the items despite assistance were excluded to safeguard data integrity; (iiii) Questionnaires were collected and the quality was controlled. Data were entered by two people for consistency check.

### Statistical analyses

All statistical analyses were conducted with the use of R software version 4.4.3 [28]. Data were first descriptively analyzed to check range and distribution of the primary variables. Correlations between continuous variables were performed using the Pearson tests, based on cases with complete data for these variables (*N* = 152). Holm’s correction was applied to p-values for multiple comparisons in the correlation matrix output. To examine for the mediating effect of premonitory urges and the moderating effect of comorbidity, we employed Bayesian Structural Equation Modeling (BSEM) implemented via the blavaan package (Version 0.5-8) using Stan (version 2.32.2) [29, 30].

In all Bayesian SEM analyses, model parameters were estimated with Markov chain Monte Carlo (MCMC) sampling. MCMC was conducted with four chains, each with 2,000 warmup iterations and 12,500 post-warmup iterations, resulting in 50,000 total posterior samples. Weakly informative priors were specified as Normal(0,10) for regression coefficients and intercepts, Gamma(1,0.5) for residual standard deviations, and Beta(1,1) for residual correlations. Model convergence was assessed using the potential scale reduction factor (Rhat), with a criterion of Rhat < 1.05 for all parameters indicating convergence. Model fit was assessed using the posterior predictive p-value (PPP), a primary method for goodness-of-fit evaluation in Bayesian analysis [31]. The fundamental principle of the PPP is that if a model provides a good fit, then data simulated from the model should resemble the observed data. The PPP quantifies this resemblance by calculating the probability that a replicated dataset, generated from the model’s posterior predictive distribution, would be more extreme than the observed dataset on a selected discrepancy statistic (e.g., a chi-square-like measure). A PPP value near 0.5 indicates a good fit, while values approaching 0 or 1 suggest model misfit, indicating that the model systematically fails to reproduce key features of the data [32]. Missing data in these models were handled using the Full Information Maximum Likelihood estimation implemented in Stan.

Our analysis proceeded in two main steps. First, a simple mediation model was tested to establish the indirect effect of YGTSS on GEC via PUTS, controlling for age, sex, and comorbidity (see Supplementary Figures S1 for details). Second, a moderated mediation analysis was conducted to investigate whether comorbidity moderated this mediation pathway. For this step, we initially constructed a comprehensive model testing moderation on all ‘a’, ‘b’, and direct effect paths (see Supplementary Tables S1 & S2). The results from this full model revealed that only the interaction term on the ‘a’ path (YGTSS × Comorbidity ->PUTS) was statistically significant (Estimate = 0.112, 95% CI [0.015, 0.210]). All other interaction terms were non-significant.

**Fig. 1:**
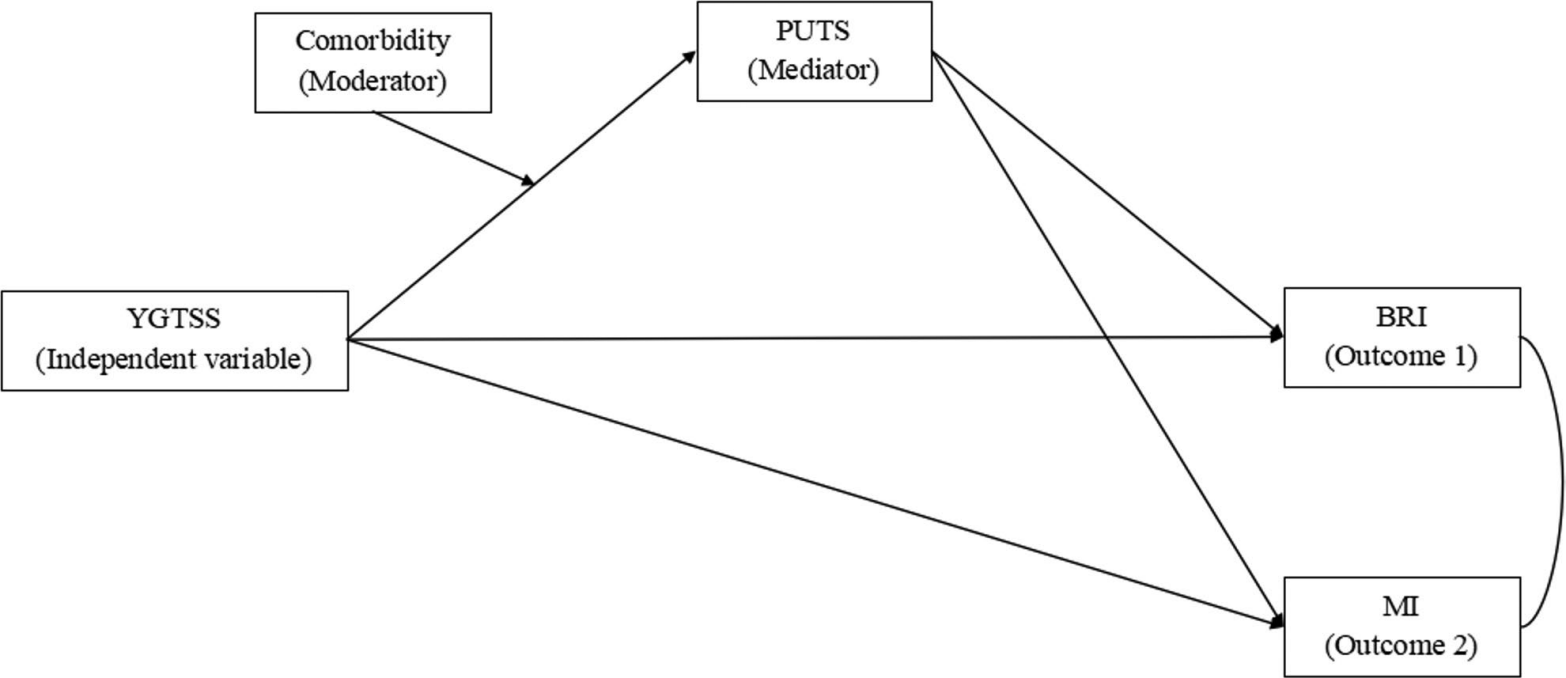
Simplified moderated mediation model with comorbid status moderating the YGTSS->PUTS path. PUTS Regression: YGTSS + Age + YGTSS×Comorbidity (‘a’ path retained); BRI/MI Regression: PUTS + YGTSS + Age + Sex + Comorbidity (direct effects only); Key Terms: YGTSS×Comorbidity = Moderation on YGTSS→PUTS (‘a’ path)

This empirical finding is consistent with clinical accounts suggesting a close interaction between tic phenomena and comorbid conditions. For instance, Eapen, Cavanna, and Robertson (2016) describe how the effort to control tics, a process intimately linked to premonitory urges, can interact with the inattention characteristic of ADHD to create a “vicious cycle” of impairment. This suggests that the presence of comorbidity may primarily alter the fundamental sensory experience of tics (the ‘a’ path) rather than changing how that experience translates into broader executive function difficulties (the ‘b’ paths) [33]. Therefore, based on this data-driven evidence, theoretical plausibility, and the principle of parsimony, we specified and adopted a more parsimonious, simplified model for our primary analyses [34]. This final model retained only the significant moderation on the ‘a’ path (see Fig. 1). The decision was further supported by the improvement in model fit, with the PPP improving from 0.000 in the full model to a more acceptable value of 0.046 in the simplified model.

## Results

### Descriptive statistics and correlation analysis

The final sample for path analyses comprised 153 participants. Descriptive statistics for the key variables are presented in Table 1. The mean total tic severity according to the YGTSS was 32.71 (SD = 13.17), mean PUTS score was 14.47 (SD = 4.58), and mean GEC raw score was 138.10 (SD = 24.34). 53.25% of the patients had at least one comorbidity.

**Table 1:** Demographic and clinical characteristics of participants

Variables	Mean (SD); *n* (%)
Sex	
Male	130 (84.42%)
Female	24 (15.58%)
Comorbidity	
Yes	82 (53.25%)
No	72 (46.75%)
Age	9.29 (1.95)
YGTSS	
Motor tic severity	11.14 (4.32)
Vocal tic severity	6.98 (5.14)
Total tic severity	32.71 (13.17)
PUTS	14.47 (4.58)
BRIEF	
BRI	47.92 (10.88)
MI	90.18 (15.38)
GEC	138.10 (24.34)

**Fig. 2:**
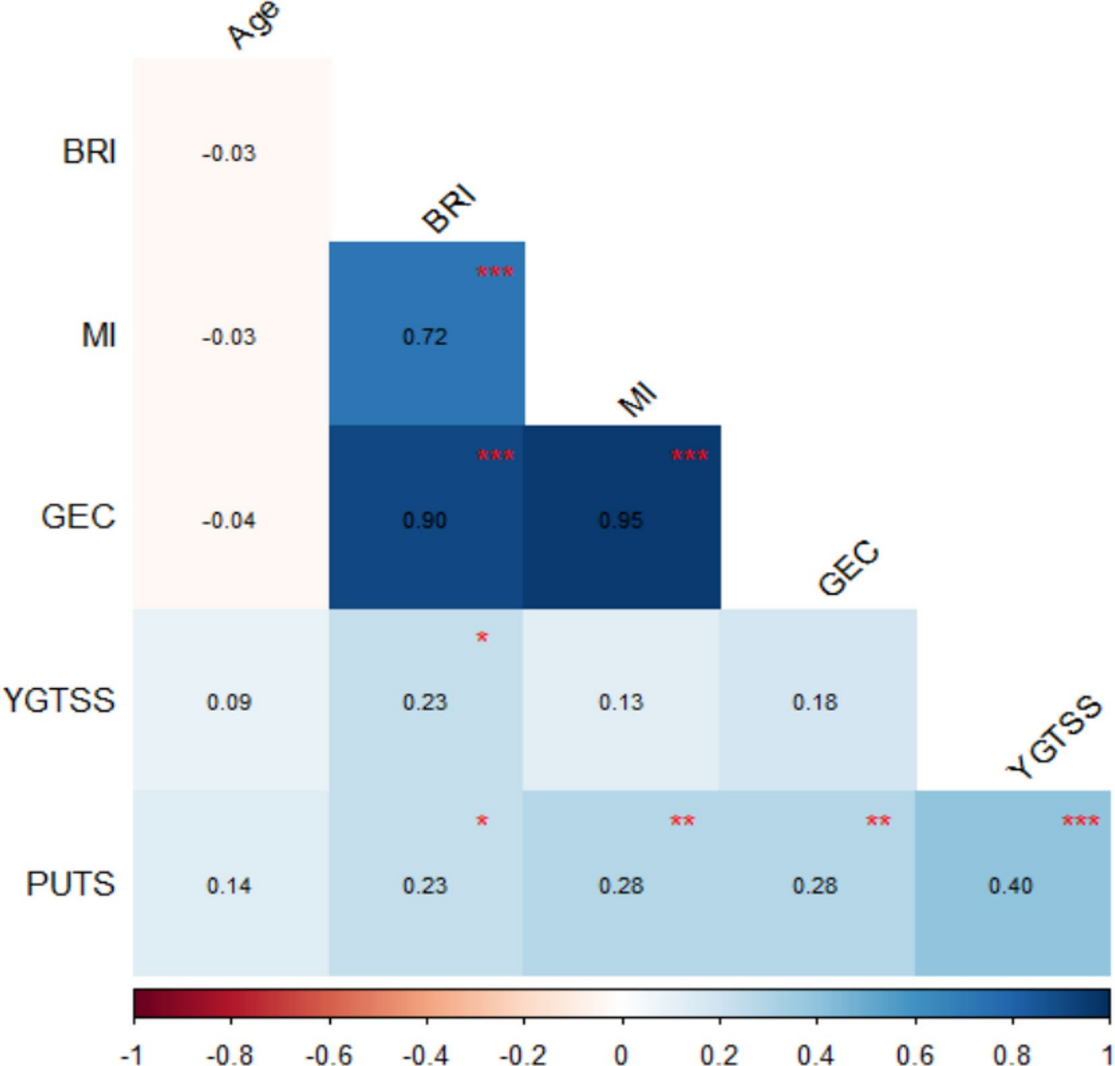
pearson correlation matrix of tic severity, premonitory urge, executive function scores, and age. Heatmap displaying pearson correlation coefficients (*r*), ordered by hierarchical clustering. Numerical coefficients are shown. Asterisks indicate statistical siginifinance based on Holm-adjusted *p-*values: * *p* < 0.005, ** *p* < 0.01, *** *p* < 0.001. Correlation analysis included *N* = 152 participants. *YGTSS* Yale Global Tic Severity Scale, *PUTS* Premonitory urge for tics Scale, *BRI* Behavioral Regulation Index, *MI* Meta cognition Index, *GEC* Global Executive Composite

Pearson correlations for continuous study variables are displayed in Fig. 2. Significant positive correlations were observed between YGTSS and PUTS (*r* = 0.40, *p* < 0.001), YGTSS and BRI (*r* = 0.23, *p* = 0.034), PUTS and BRI (*r* = 0.23, *p* = 0.033), PUTS and MI (*r* = 0.28, *p* = 0.005), and PUTS and GEC (*r* = 0.28, *p* = 0.005). As expected, BRI, MI, and GEC were strongly intercorrelated (*r* = 0.72 ~ 0.95, *p* < 0.001). Age was not significantly related to other variables.

### Simple mediation model (YGTSS ->PUTS ->GEC)

Model fit indices all indicated that the Bayesian simple mediation model provided a good fit to the data (PPP = 0.512) and convergence (all Rhats < 1.05). As shown in Supplementary Table S3 and S4, the path from YGTSS to PUTS (‘a’ path) was significant (Estimate = 0.130, 95% CI [0.081, 0.180], β = 0.377). The path from PUTS to GEC (‘b’ path) was significant (Estimate = 1.082, 95% CI [0.209, 1.960], β = 0.205). The direct effect of YGTSS on GEC (c’ path) was not significant (Estimate = 0.188, 95% CI [-0.105 0.481], β = 0.103). Notably, there was a significant indirect effect of YGTSS on GEC via PUTS (Estimate = 0.141, 95% CI [0.013, 0.269], β = 0.077). The standardized coefficient suggests that for each standard deviation increase in tic severity, an indirect increase of 0.077 standard deviations in global executive function difficulties is transmitted through the premonitory urge. Comorbidity significantly predicted both PUTS (Estimate = 1.644, 95% CI [0.331, 2.957], β = 0.181) and GEC (Estimate = 10.992, 95% CI [4.114, 17.795], β = 0.229), whereas neither age nor sex were significant predictors. These results support a full mediation model where the effect of tic severity on overall executive function difficulties is fully transmitted through the premonitory urge, after controlling for covariates.

### Moderated mediation model (Simplified ‘a’ path only, predicting BRI and MI)

The final simplified moderated mediation model, focusing on the ‘a’ path moderation for outcomes BRI and MI, demonstrated improved model fit statistics relative to the full model (PPP = 0.046). As shown in Table 2, the main effect of YGTSS on PUTS (a1) was significant (Estimate = 0.094, 95% CI [0.041,0.148], β = 0.272). Importantly, the interaction term YGTSS × Comorbidity (a4) was also significant (Estimate = 0.061, 95% CI [0.025, 0.098], β = 0.262), indicating that the positive association between tic severity and the premonitory urge was significantly stronger in the comorbid group. PUTS significantly predicted higher scores on both BRI (b1: Estimate = 0.592, 95% CI [0.178, 1.018], β = 0.224) and MI (c1: Estimate = 1.175, 95% CI [0.611, 1.761], β = 0.308). YGTSS showed a significant direct effect only on BRI (b2: Estimate = 0.196, 95% CI [0.056, 0.337], β = 0.214), but not on MI (c2: Estimate = 0.127, 95% CI [-0.065, 0.322], β = 0.096). Comorbidity independently predicted higher scores on both BRI (b5: Estimate = 4.095, 95% CI [0.675, 7.516], β = 0.170) and MI (c5: Estimate = 8.343, 95% CI [3.608, 13.072], β = 0.240). Age significantly predicted higher MI scores (c3: Estimate = 1.249, 95% CI [0.136, 2.401], β = 0.140) but not BRI scores. Sex had no significant effect on either outcome.

**Table 2:** Bayesian path analysis results for the simplified moderated mediation model

Parameter / Path	Estimate (Mean)	Post.SD	95% CI	Sig	Std.all (β)	Rhat
Predicting PUTS (Mediator)						
YGTSS ->PUTS (a1)	0.094	0.027	[0.041, 0.148]	*	0.272	1.000
Age ->PUTS (a2)	0.207	0.166	[-0.122, 0.532]		0.089	1.000
YGTSS×Comorbidity ->PUTS (a4)	0.061	0.019	[0.025, 0.098]	*	0.262	1.000
Predicting BRI (Outcome 1)						
PUTS ->BRI(b1)	0.592	0.214	[0.178, 1.018]	*	0.224	1.000
YGTSS ->BRI (b2)	0.196	0.072	[0.056, 0.337]	*	0.214	1.000
Age ->BRI (b3)	0.800	0.427	[-0.024, 1.650]		0.129	1.000
Sex ->BRI (b4)	1.043	2.278	[-3.398, 5.527]		0.031	1.000
Comorbidity ->BRI (b5)	4.095	1.749	[0.675, 7.516]	*	0.170	1.000
Predicting MI (Outcome 2)						
PUTS ->MI (c1)	1.175	0.293	[0.611, 1.761]	*	0.308	1.000
YGTSS ->MI (c2)	0.127	0.099	[-0.065, 0.322]		0.096	1.000
Age ->MI (c3)	1.249	0.579	[0.136, 2.401]	*	0.140	1.000
Sex ->MI (c4)	-2.179	3.130	[-8.239, 3.998]		-0.046	1.000
Comorbidity ->MI (c5)	8.343	2.394	[3.608, 13.072]	*	0.240	1.000
Residual Variances						
Residual Var (PUTS)	16.081	1.855	[12.856, 20.090]	*	0.777	1.000
Residual Var (BRI)	114.014	13.664	[90.146, 143.679]	*	0.786	1.000
Residual Var (MI)	218.552	26.606	[172.164, 276.459]	*	0.726	1.000
Residual Covariance						
Residual Cov (BRI ~ ~ MI)	111.551	16.399	[82.612, 146.918]	*	0.707	1.000

Estimate = Posterior Mean; Post.SD = Posterior Standard Deviation; 95% CI = 95% Equal-Tailed Credible Interval; Sig = Significance marker (* indicates 95% CI excludes 0); Std.all (β) = Fully standardized coefficient; Rhat = Potential Scale Reduction Factor. YGTSS = Yale Global Tic Severity Scale; PUTS = Premonitory Urge for Tics Scale; BRI = Behavioral Regulation Index; MI = Metacognition Index; Comorbidity (0 = No, 1 = Yes); Sex (0 = Male, 1 = Female); Age (years). Model includes moderation on the ‘a’ path (YGTSS×Comorbidity ->PUTS) only. Intercepts are not reported. PPP for this model was 0.046. *N* = 153

Conditional indirect effects and the index of moderated mediation (IMM) are presented in Table 3. The indirect effect of YGTSS on both BRI and MI via PUTS was significant for both the non-comorbid group (BRI: Estimate = 0.056, 95% CI [0.004, 0.108], β = 0.061; MI: Estimate = 0.111, 95% CI [0.027, 0.195], β = 0.084) and the comorbid group (BRI: Estimate = 0.092, 95% CI [0.020, 0.164], β = 0.119; MI: Estimate = 0.183, 95% CI [0.075, 0.290], β = 0.165). Notably, the Index of Moderated Mediation (IMM) was significantly positive for both outcomes (BRI: Estimate = 0.036, 95% CI [0.002, 0.070], β = 0.059; MI: Estimate = 0.072, 95% CI [0.016, 0.128], β = 0.081). This confirms a significant moderated mediation effect. Clinically, this finding is important as it quantifies the heightened risk for the comorbid subgroup; for instance, the indirect effect of tic severity on metacognition (MI) is nearly twice as strong for children with comorbidities (β = 0.165) as for those without (β = 0.084). Specifically, comorbidity significantly strengthened the indirect effect of tic severity on both behavioral regulation (BRI) and metacognition (MI) difficulties through the pathway of the premonitory urge, driven by its moderation of the YGTSS-PUTS relationship (‘a’ path).

**Table 3:** Conditional indirect effects and index of moderated mediation for the simplified model

Defined Parameter (Term)	Estimate (Mean)	Post.SD	95% CI	Sig	Std.all (β)
Indirect Effect (BRI | Comorbidity = 0)	0.056	0.026	[0.004, 0.108]	*	0.061
Indirect Effect (MI | Comorbidity = 0)	0.111	0.043	[0.027, 0.195]	*	0.084
Indirect Effect (BRI | Comorbidity = 1)	0.092	0.037	[0.020, 0.164]	*	0.119
Indirect Effect (MI | Comorbidity = 1)	0.183	0.055	[0.075, 0.290]	*	0.165
Index of Mod. Med. (BRI)	0.036	0.017	[0.002, 0.070]	*	0.059
Index of Mod. Med. (MI)	0.072	0.029	[0.016, 0.128]	*	0.081

Estimate = Posterior Mean; Post.SD = Posterior Standard Deviation; 95% CI = 95% Equal-Tailed Credible Interval; Sig = Significance marker (* indicates 95% CI excludes 0); Std.all (β) = Fully standardized coefficient; Index of Mod. Med. = Index of Moderated Mediation testing the difference in indirect effects between comorbidity groups. *N* = 153

## Discussion

In this study, we demonstrated through a moderated mediation model that tic severity indirectly affects executive functioning (including BRI and MI) through the premonitory urge. Additionally, our findings indicated that comorbidity significantly strengthened the association between tic severity and executive functioning by influencing the pathway from tic severity to the premonitory urge. This finding offers a novel theoretical framework for comprehending the neurobehavioral mechanisms underlying tic disorders and identifies a potential target for clinical intervention.

### Relationship between tic severity and premonitory urges

As one of the core characteristics of tic disorders, premonitory urges and tics are coupled in real time. However, the severity of premonitory urges evaluated by clinical scales is not consistent with the severity of tics, and some clinical scales show no significant correlation between them [35]. This study found that children with higher YGTSS scores also have higher PUTS scores, which is consistent with many studies [14, 15]. That is, patients with more severe tics tend to report more pronounced premonitory urges. This association may be supported by neuroimaging studies, which suggest that regions such as the precuneus and posterior cingulate cortex (pCunPCC) could play a key role in mediating the relationship between premonitory urges and tic severity [36], but further validation is needed.

### Effect of premonitory urges on executive function

The present study found significant effects of premonitory urges on executive functioning, including BRI and MI. From a neural mechanism perspective, it is hypothesized that dysfunction of the supplementary motor area (SMA) and insula might be involved in this process. Some evidence indicates that abnormalities in GABA^+^ levels in SMA could be correlated with the strength of the premonitory urge [37]. Excessive activation in SMA brain regions might contribute to enhanced perception of premonitory urges and impaired motor inhibition function (reflected in BRI deficits) [38, 39], and concomitantly decreased ability of insula to integrate sensory information (reflected in MI impairments) [40]. However, these mechanisms remain speculative without direct empirical support from this dataset.

### Mediating role of premonitory urges between tic severity and executive function

We found that children and adolescents with more severe tics had more impairments in executive function, which is consistent with previous studies [10–13]. Notably, the direct effect of tic severity on executive function was found to be weak in the present study, while the indirect effect through the premonitory urge was significant. This finding suggests that premonitory urge may act as a mediating variable, potentially transforming motor symptoms into cognitive dysfunction. This mediating effect was also found to be significant in both groups with and without comorbidities.

The clinical and practical significance of this mediating pathway is a central finding of our study. The standardized indirect effect of YGTSS on GEC in our simple mediation model was β = 0.077. While this might be considered a “small” effect by conventional heuristics, such labels can be misleading without context [41]. In the field of child development, a small but persistent mechanistic pathway can have substantial cumulative consequences over time. As Zelazo and Carlson (2020) have argued, early deficits in executive function (EF) can impact a child’s readiness to learn, potentially widening academic achievement gaps throughout their schooling [42]. A consistent, mediated increase of nearly one-tenth of a standard deviation in EF difficulties, linked to tic severity, could therefore translate into meaningful differences in a child’s academic and social trajectory.

The finding of this mediating effect suggests that interventions targeting premonitory urges may not only improve tic symptoms but may also indirectly alleviate executive function impairments. Comprehensive Behavioral Intervention for Tics (CBIT) and Habit Reversal Training (HRT), for example, may simultaneously reduce tic symptoms and improve executive functioning by helping patients identify premonitory urges and develop alternative behaviors. In addition, assessing premonitory urges in children with tic disorders may help predict the risk of executive function impairment and provide a basis for early intervention.

### Comorbidity strengthened the effect of tic severity on premonitory urges

This study found the moderating role of comorbidities in the pathway between tic severity and premonitory urges; that is, comorbidities could enhance the effect of tic severity on premonitory urges. The findings align with previous studies. The comorbidity of ADHD might influence the associations between premonitory urges and tics, possibly through the dopaminergic system. In patientswith comorbid ADHD, dysregulated dopamine signaling may be linked to sensorimotor gating deficits, potentially impairing the ability to filter somatic sensations and thus amplifying premonitory urges [43]. Studies in children, adolescents, and/or adults with tic disorders have observed a significant association between the severity of premonitory urges and OCD symptoms [3, 4, 35, 44]. OCD symptoms, particularly “not-just-right experiences” (NJREs), may enhance the perception of premonitory urges [45]. In addition, the introduction of the concept of “Tourettic OCD” further illustrates that comorbid conditions may affect both tics and premonitory urges through shared neural mechanisms, such as abnormalities in the prefrontal-striatal loop [46]. Comorbid conditions, including intellectual disability, may make it more difficult for individuals to suppress premonitory urges due to cognitive limitations [47, 48]. It is critical to emphasize that these proposed neurobiological mechanisms, while plausible and aligned with the existing literature, remain hypothetical and require direct empirical validation in future studies.

The clinical utility of this moderation effect is substantial. Our analysis revealed that the magnitude of the indirect effect of tic severity on metacognition (MI) was nearly twice as strong for children with comorbidities (β = 0.165) compared to those without (β = 0.084). This difference is not only statistically robust but also clinically meaningful, as it identifies the comorbid subgroup as being particularly vulnerable to the indirect impact of tics on higher-order cognitive skills. This insight provides a strong rationale for stratified care approaches.

Consequently, these findings indicate that systematic screening for comorbidities should be incorporated into future clinical evaluations of tic disorders. Treatment decisions should account for the influence of comorbidities on the symptom network, necessitating tailored intervention programs for different comorbidity profiles. When applying these insights, the cultural and clinical context of our Chinese tertiary hospital sample is a crucial consideration. Help-seeking behaviors and cultural norms (e.g., around discipline or describing internal experiences) may influence symptom reporting on instruments like the BRIEF and the expression of premonitory urges. While our model identifies robust associations, the effective translation of our findings into other settings will benefit from future research that tests this model across diverse populations, using our work as a foundational benchmark.

## Limitations

However, it is essential to acknowledge the limitations of this study. First, the study was a cross-sectional design, and although associations between variables were revealed through mediation and moderation analyses, causality could not be rigorously established. Future research should aim to verify the directionality of these relationships using a longitudinal design tracking design or experimental intervention studies (e.g., changes in executive functioning after an intervention treatment targeting premonitory urges). Second, our measurement approach, while robust in its ecological validity, has inherent constraints. The assessment of executive function relied solely on parent-reported ratings (BRIEF), which is susceptible to informant bias. The psychological state and cultural expectations of parents may influence their perceptions, potentially affecting the precision of estimated relationships with other variables. Similarly, the self-reported nature of the PUTS scale, despite our standardized assistance protocol, may have introduced measurement error, particularly in younger children. Furthermore, treating comorbidities as a collective moderator precluded the examination of disorder-specific effects (e.g., ADHD vs. OCD). Consequently, future studies would benefit from a multi-method, multi-informant approach, incorporating objective neuropsychological tests (e.g., Stroop) and teacher reports, among others. Third, the generalizability of our findings is limited by sample characteristics. There was a significant gender imbalance (130 boys vs. 24 girls), which reflects the TD population but restricts analysis of sex-specific effects and reduces applicability to females. Moreover, participants were recruited from a single tertiary medical center in China. Therefore, the portability of our moderated mediation model requires future validation in more diverse and representative populations.

## Conclusion

In conclusion, this study reveals that the premonitory urge mediates the relationship between tic severity and executive functioning in childhood tic disorders and that comorbidity moderates this mediating effect. It is found that the premonitory urge, a key mediator linking motor symptoms to cognitive dysfunction, is modulated by comorbidity, which in turn exacerbates executive function impairment. This finding provides a new perspective for understanding the multidimensional clinical manifestations of tic disorders and a theoretical basis for optimizing clinical assessment and intervention strategies. Further validation of causal mechanisms in combination with longitudinal design and neuroimaging is needed in the future, and more precise and individualized intervention protocols need to be explored.

## Supplementary Information

Below is the link to the electronic supplementary material.

Supplementary Material 1.

## Data Availability

No datasets were generated or analysed during the current study.

## Abbreviations

YGTSS: The Yale Global Tic Severity Scale
PUTS: Premonitory Urge for Tics Scale
BRIEF: Behavior Rating Inventory of Executive Function
BSEM: Bayesian Structural Equation Modeling
BRI: Behavioral Regulation
MI: Metacognition
TS: Tourette syndrome
ADHD: Attention Deficit/hyperactivity Disorder
OCD: Obsessive-compulsive Disorder
ASD: Autism SpectrumDisorder
DSM-5: Diagnostic and Statistical Manual of Mental Disorders, Fifth Edition
GEC: Global Executive Composite
